# Genomic Data Suggests Pathways of Modern White Poplar (*Populus alba* L.) Range Formation in the Postglacial Era

**DOI:** 10.3390/plants14213328

**Published:** 2025-10-30

**Authors:** Natalya S. Gladysh, Mikhail I. Popchenko, Maxim A. Kovalev, Vsevolod V. Volodin, George S. Krasnov, Alina S. Bogdanova, Dmitry S. Karpov, Nadezhda L. Bolsheva, Anna V. Kudryavtseva

**Affiliations:** 1 Engelhardt Institute of Molecular Biology, Russian Academy of Sciences, 32 Vavilova, Moscow 119991, Russia; natalyagladish@gmail.com (N.S.G.); popchenko_m@inbox.ru (M.I.P.); vsevolodvolodin@yandex.ru (V.V.V.); gskrasnov@mail.ru (G.S.K.); alina.bogdashka@yandex.ru (A.S.B.); aleom@eimb.ru (D.S.K.); nlbolsheva@mail.ru (N.L.B.); rhizamoeba@mail.ru (A.V.K.); 2 Institute of Geography, Russian Academy of Sciences, Staromonetny Pereulok, 29/4, Moscow 119017, Russia; 3 Center for Precision Genome Editing and Genetic Technologies for Biomedicine, Engelhardt Institute of Molecular Biology, Russian Academy of Sciences, Vavilov Str., 32, Moscow 119991, Russia; 4 Faculty of Biology and Biotechnologies, National Research University Higher School of Economics, Myasnitskaya Str., 20, Moscow 101000, Russia

**Keywords:** admixture, Eurasia, glaciers, historical formation, natural habitat, *Populus alba*, quaternary glaciation, SNP profiling, whole-genome sequencing

## Abstract

The white poplar (*Populus alba* L.) is an economically significant tree species with a natural distribution spanning an extensive region of Eurasia. Nevertheless, there is currently no hypothesis regarding the historical shaping of this range. In this study, we collected and sequenced 36 individuals of white poplar from disparate regions of Russia and Kazakhstan. Additionally, we employed available genomic data of white poplars from Italy, Hungary, and China. A genomic approach was employed to collate data on the location of glaciers in different periods, along with information on the natural and artificial distribution of white poplar. This enabled the formulation of the first hypothesis regarding the formation of the modern range of this plant. It is hypothesized that during the period of maximum glaciation, three refugia existed: the South European, Transcaucasian, and Altai–Middle Asian refugia. Postglacial migration from these refugia led to the formation of modern populations of *P. alba* in Eastern Europe (including the European part of Russia), the Caucasus, and Siberia, respectively.

## 1. Introduction

The family Salicaceae separated about 128 million years ago. It includes 54 genera and about 1400 species, with most genera represented by a small number of species and distributed in Southeast Asia, the likely center of origin of the family [1]. The most evolutionarily successful genera are *Salix* and *Populus*. Their common ancestor, about 60–65 million years ago, underwent Salicoid whole-genome duplication, which affected approximately 92% of the genome and resulted in more than 8000 pairs of paralogous genes [2]. This is probably why the genera *Salix* and *Populus* were able to spread over almost all of the Northern Hemisphere, especially in the boreal regions, and now number around 450 and 50 species, respectively [3,4]. Interestingly, modern species of the genus *Populus* began to diversify in the early Oligocene, approximately 27.24 mya. Three independent lines left Eurasia and colonized North America across the Bering Sea bridges, which occurred ~60, ~24, and ~10 mya [5].

The genus *Populus* is divided into four sections: Abaso, Turanga, Populus, and ATL, the latter including representatives of the traditional sections Aigeiros, Tacamahaca, and Leucoides. Abaso is the most primitive section, including *P. mexicana*, which is common in Mexico. Turanga includes *P. euphratica*, known to grow in deserts in the Middle East and named after the Euphrates River. Populus and ATL are the most evolutionarily advanced groups. Populus includes the aspens *P. tremula* and *P. tremuloides*, as well as *P. alba*, *P. tomentosa*, *P. qiongdaoensis*, and some other poplars. The ATL group includes *P. trichocarpa*, *P. balsamifera*, *P. deltoides*, *P. nigra*, *P. lasiocarpa*, and others [6]. Interestingly, poplars, like willows, are dioecious plants, meaning that individual trees are either male or female, which is a much less common situation in the plant world compared to monoecious plants, where both male and female reproductive structures can be found on the same individual. Some representatives of the Populus section, including *P. alba*, have a ZW system of sex determination, while most poplars are characterized by an XY system [7,8]. Moreover, only two species are tropical in the genus *Populus*, namely, *P. qiongdaoensis* and *P. ilicifolia*, distributed in Hainan and Africa (Kenya and Tanzania), respectively [8]. *P. trichocarpa* and *P. balsamifera* are distributed in temperate forests of North America. *P. trichocarpa* is spread along the west coast of the United States and Canada, while *P. balsamifera* occurs somewhat northward, from Alaska to Labrador; however, there is a significant degree of hybridization between these closely related species in the habitat overlap zone [9,10,11,12]. *P. tomentosa* is a Chinese endemic [13].

White poplar (*Populus alba*) is a well-known member of the genus. Its natural range extends from Western and Southern Europe to Altai, Xinjiang, and the Western Himalayas, bounded in the north by mixed forests and taiga, where the climate is too cold for it, and in the south by steppes, semi-deserts, and deserts, where it is too dry. *P. alba* did not spread across the Altai, probably due to the harsh, sharply continental climate of Eastern Siberia and competition from other species [14,15,16,17]. However, due to its desirable biological characteristics, *P. alba* was introduced to many countries, and it can now be found on all five continents (https://powo.science.kew.org/taxon/urn:lsid:ipni.org:names:776573-1, accessed 13 October 2024).

As for the economic point of view, poplars have pronounced economic value. They have long been used as a source of pulp and wood, and the International Poplar Commission (IPC) was established in 1947 to promote the sustainable management of fast-growing trees by its 38 member countries [18]. At the same time, due to their fast growth, poplars can be used to combat climate change, as they store atmospheric carbon in their wood [19,20]. Poplars have traditionally been used for landscaping rural and urban areas, including in Russia. This is partly due to their ability to withstand heavy soil pollution, which allows poplar to be used for phytoremediation, cleaning both urban and industrial soils [21,22]. Finally, poplars are used by scientists as model plants among trees—for example, *Populus trichocarpa* became the first tree whose genome was sequenced in 2006 [2]. Due to all these reasons, the geographical distribution of selected valuable poplar species has become wider through introduction in selected regions.

The complex history of white poplar range formation, including the reduction in its habitat area during the glacial period and the subsequent development of new territories, has led to the formation of significant intraspecific genetic diversity, which arose both because of range disjunction and the need to adapt to the conditions of very diverse regions in terms of climate and biota. Populations from different regions are characterized by very contrasting indicators of growth rate, winter hardiness, frost resistance, drought tolerance, and salt tolerance, as well as resistance to pests and pathogens, primarily those that cause stem rot. In addition, populations of different origins differ in their ability to vegetatively reproduce and the set of symbiotic microorganisms, which is very important in connection with the increasing use of biotechnology methods in the cultivation of these tree species. The current intraspecific diversity has both great practical applications in breeding work and can be used to reconstruct the history of white poplar dispersal after the retreat of glaciers; to identify primary and secondary ranges of the species, the history of its origins, and the dispersal of individual forms; and to solve problems of intraspecific systematics [17,23,24,25,26].

In this study, we present the first insights into the formation of the modern *P. alba* region during the postglacial era, based on the genomes of 36 white poplar individuals collected in Russia and Kazakhstan, and using available genomic data of white poplars from Italy, Hungary, and China. Our findings shed light on the evolutionary history of this species in the East European Plain, the Caucasus, and Western Siberia. The data may also be employed to model the range dynamics of other species of the genus in Eurasia and North America, as poplars exhibit analogous strategies in the development of their populations.

## 2. Results

**Figure 1:**
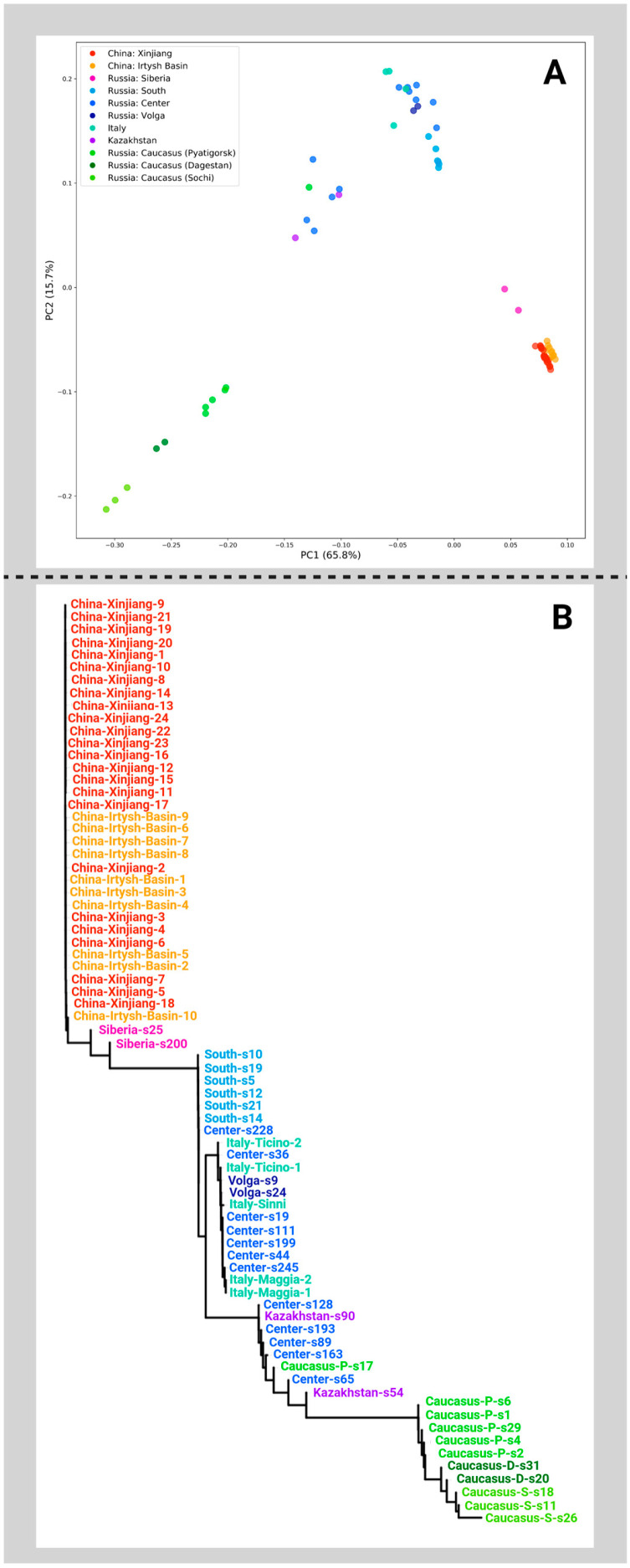
(**A**) PCA plot based on genotype likelihood analysis. Three major clusters can be seen, the first of which corresponds to samples from China (Xinjiang Province and Irtysh River Valley) and Western Siberia (Novosibirsk); the second to plants from the Caucasus (Sochi, Pyatigorsk, and Dagestan); and the third to all other samples, coming from Italy and the European part of Russia. (**B**) NJ phylogenetic tree corresponding to this PCA plot. Three–four clusters are also visible.

To understand the process of modern white poplar range formation, we used a genomic approach. To do so, we derived genomic data from plants originating from a range of countries and geographic locations and identified clusters of genetically similar samples. Based on the genotype likelihoods, we performed PCA analysis (the results were similar). Principal components 1 and 2 totally explained > 80% of the total variations, as can be seen in Figure 1A. The corresponding NJ phylogenetic tree is shown in Figure 1B. Both a PCA plot and an NJ tree were produced by PCAngsd. Similar results were derived for SNP data (a total of about 23 million SNPs present in at least one sample were identified).

Additionally, we inferred a UPGMA tree based on SNP profiles (Supplementary Figure S1) and a maximum likelihood tree (also based on SNP data, for coding regions), which is presented at Supplementary Figure S2. Overall, the topology of all trees shares common fundamental features, and we can speak of a sufficient degree of consistency in the obtained data.

**Figure 2:**
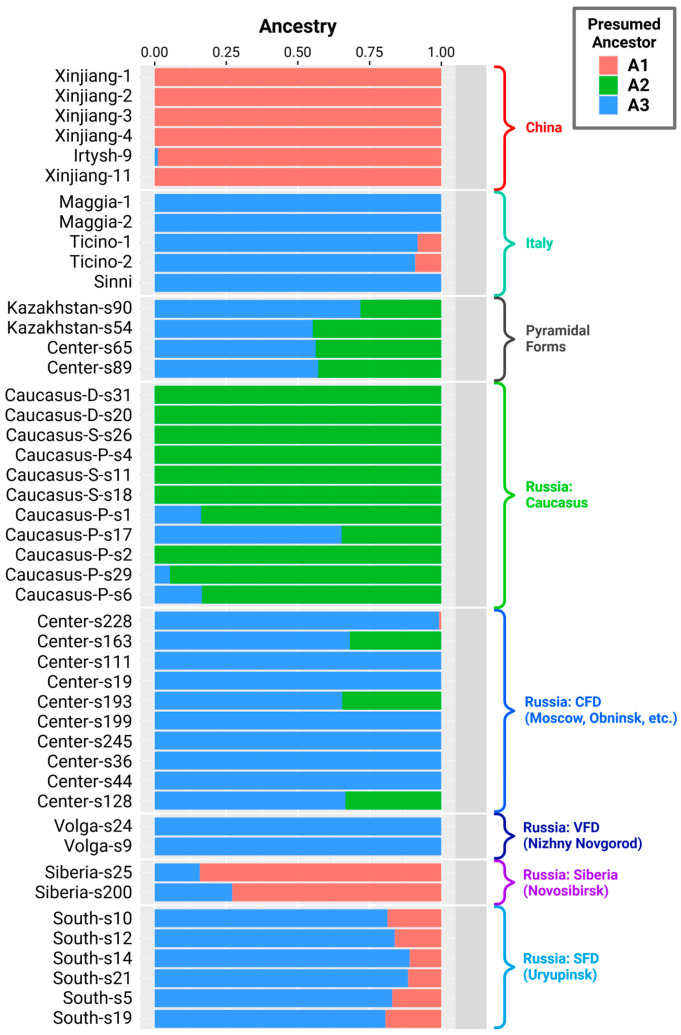
Results of the population admixture analysis with three (K = 3) presumable ancestors (A1, A2, A3). A1 is the most likely ancestor of Chinese poplars; A2 of Caucasian poplars; and A3 of Italian plants, as well as poplars growing in Central Russia. The Siberia-s13 sample is omitted because it is a *P. alba* × *P. tremula* hybrid.

Based on the PCA plot and phylogenetic trees, we identified three clusters. First, samples collected in the Caucasus (in the vicinity of Sochi and Pyatigorsk and in Dagestan) were most strongly separated from all other samples and formed a separate cluster. Chinese (growing in Xinjiang and the Irtysh River valley) and Siberian trees were also grouped together. All other plants formed a continuous third cluster. Noteworthily, the Italian specimens were the furthest away, as were plants from Beijing (which were most likely introduced to China). Finally, six poplars, four of which were pyramidal (these are all pyramidal poplars in our study), formed a separate subcluster inside this third cluster. The same clusters of samples are clearly visible in the dendrogram presented in Supplementary Figure S1. Thus, we hypothesized that there were probably three ancestral populations according to the three identified clusters. Based on these genotypes, the entire modern diversity of white poplars could be formed. To test this hypothesis, we studied the genetic admixture structure with NGSAdmix and other tools, as written in the Materials and Methods section. The results are presented in Figure 2.

We ran NGSAdmix with various numbers of presumable ancestors (K, from two to six), and the results are shown in Figure S5. For K = 2 and 3, the most stable structures were observed; they were similar when different initial seeds were used. Also, various tools produced similar structures at K = 2 or 3, as can be seen in Figure S6 (ADMIXTURE) and Figure S7 (PCangsd). At K = 4 and above, the inferred structures differed significantly from one to another, both between various tools and between various seeds. The most significant differences were observed in the Central region, which was classified (according to ‘admixture’ results) either as a mixture of haplotypes or as a single haplotype. At K = 4, the variations were also observed in the Chinese samples. According to the ‘admixture’ results (NGSAdmix does not allow for CV error estimation), the cross-validation errors were minimal at K = 2 and 3 (Figure S8). At K = 3, the derived genetic split corresponds to geography (Europe, Caucasus, and Chinese regions). Also, when K > 3, a cluster of samples appears with ‘pure’ haplotypes in the Southern Federal District, where it is unlikely that a refugium existed during glaciation due to the harsh climatic conditions.

Nevertheless, the data obtained at K = 2 allow us to conclude that Chinese and Caucasian poplars are the most distant from each other, and the separation of samples from China (observed in some variants of the analysis at K = 4) allows us to hypothesize that in the mountains of Xinjiang and Altai, there could be several, with at least two, distinct populations.

We interpreted the results of this analysis as follows. A1 is the most probable ancestor of poplars in China, largely in Western Siberia, and has also contributed to the gene pool of plants in the vicinity of Uryupinsk. A2 is a key ancestor of trees in Transcaucasia and the Caucasus, and its participation in the artificial settlement of the Central Russia territories and the creation of pyramidal forms is also notable. A3 is an ancestor of poplars from Italy, a key member of the gene pool of the central and lower Volga regions, a secondary area of the central part of Russia, and also contributed to populations in southwestern Siberia and the Caucasus and the creation of pyramidal forms, known as the variation *Pyramidata sovietica*. We reveal our theory of white poplar distribution in more detail in the Discussion section.

## 3. Discussion

We did not find articles describing a possible scenario for the formation of the whole modern range and population structure of *P. alba*, but there were several articles devoted to particular regions of the World. For example, it was shown that the two main refugia in Central and Southern Europe during glaciation were in Italy and Romania. At the same time, *P. tremula* survived in more northerly and harsh conditions, near the ice shield of the glaciated Alps. When the glaciers retreated, the ranges of both species expanded, and in the contact zone, they began to hybridize actively, forming the natural hybrid *P. × canescens* [27]. In another paper, Fussi et al. demonstrated that white poplars in Malta exhibit relatively low genetic diversity, are related to Italian trees, and also have connections to North African poplars. Moreover, it has been suggested that it is most likely that *P. alba* was introduced to Malta by humans in the sixteenth century, rather than having originally lived there [28]. On the other hand, white poplars in China, growing in the Irtysh River basin, have also been studied. Interestingly, the heterozygosity of populations there is lower than in Italy and Hungary [29]. In addition, in this region, as in Europe, *P. alba* also hybridized significantly with *P. tremula*. This process, in turn, was significantly limited by plastid–nuclear incompatibility [30].

We will also consider analogous research for two other species, namely, *P. cathayana* in China and *P. balsamifera* in Canada and the USA, just for example.

A recent study of *P. cathayana* revealed four genetically distinct populations, named by the authors for growing in Southwest (SW), Northwest (NW), and North China (NC) and in the Taihang Mountains (TH). Splitting of the ancestral population began 1649 thousand years ago (kya), with one branch dividing 1430 kya into TH and NW, and the other branch splitting 987 kya into NC and SW. The distribution and adaptation of *P. cathayana* have been linked to climate change [31].

Another interesting evolutionary story concerns the postglacial distribution of *P. balsamifera* in North America after the last glacial maximum, which occurred about 18,000 years ago. The authors identified three demes, namely, the Central, Northern, and Eastern ones. The Central deme is the most genetically diverse, occupies the largest area, and is probably directly related to the ancestral population in the refugium. The Northern deme occupies Alaska, and the Eastern deme inhabits Quebec and Labrador [11]. In another paper, it was shown that the boundary between the Eastern and Central demes is clearer than between the Central and Northern demes (there referred to as Western), which can probably be explained by the earlier separation of the Eastern deme from the general evolutionary branch [12].

Quaternary glaciation appears to have been a significant force in shaping the present-day range of *P. alba*. It began 2.58 million years ago and has continued to modern times, with periods of glaciation alternating with much warmer periods of interglaciation [32,33]. For example, the last glaciation ended 11,700 years ago, followed by the Holocene, which is an interglacial period [34,35], and the next glaciation is projected to begin in 50,000 years [36]. During the maximum glaciation, glaciers were in the northern part of Europe (most of Great Britain; the territories of modern Germany, Poland, and Belarus; northern Ukraine; and part of Russia); also, the centers of glaciation were mountain systems: the Alps and the Caucasus. However, in Eastern Siberia, glaciation also occurred but covered quite a small percentage of all surfaces—the dry continental climate probably did not allow for the formation of a glacier [37,38,39].

As for some paleontological data on *P. alba*, unfortunately, its predominantly plain modern range in Russia and ecotopic preferences determine a very poor paleontological record with its participation. Relatively few paleontological finds of Populus alba itself and species of its closest relatives, often included in its composition or species that can be considered ancestral to modern *Populus alba*, are known in the former USSR. Most of these finds were made in the Caucasus, much less in the European part of Russia, and there are no known finds from Western Siberia or Central Asia [40].

Finds identified as *Populus alba* are known in the Caucasus from the Pliocene of Sulak (Dagestan), Upper Pliocene of Sukhumi and Lechkop (Abkhazia), Upper Pliocene–Lower Pleistocene of Shamba (Armenia), and Middle Pleistocene of Kyzyl-Burun (Azerbaijan). Those from the Upper Pliocene–Lower Pleistocene of Shamba (Armenia) are also known specimens identified as *Populus canescens* (Ait.) Smith. (*P. hybrida* Bieb.) (under the name *P. canescens* (Ait.) Smith. (*P. hybrida* Bieb.)); this refers to forms close to the species *P. alba*, currently widespread in Transcaucasia. In addition, *Populus gokhtuniae* of I. Gabrielyan, close in morphology to *P. alba*, has been found in the Upper Pliocene–Lower Pleistocene of Shamba (Armenia). It is close in morphology to forms of white poplar from Central Asia known as *Populus bachofenii* Wierzb. Ex Rochel. There are known finds of Populus pliobolleana Kolak. from the Upper Miocene of Pshekha (Pre-Caucasus) and the Lower Pliocene of Kador, Bagazhisht, and Duab (Abkhazia), which have morphological similarity to modern white poplars of both the Pre-Caucasus and Transcaucasia [40].

*Populus kolesnikovae* Iljinskaja, morphologically close to both the modern white poplar and the fossil *P. pliobolleana* Kolak. but differing from it owing to leaves with width prevailing over length, was described from the Upper Miocene–Lower Pliocene of Mayachny (Chelyabinsk Oblast, Urals, Russia). *Populus amplifolia* Pimen, similar to *P. germanica* (Menzel) Walther and barely distinguishable from modern white poplar, has been described from the Upper Oligocene of Shesterintsy (Cherkassy Oblast, Ukraine). Finally, from the Upper Eocene of Tim (Kursk Oblast, European part of Russia), there are known finds similar to modern *Populus alba*, defined as *Populus germanica* (Menzel) Walther and the recently described *Populus eichwaldii* (Palib.) Vikulin [40].

**Figure 3:**
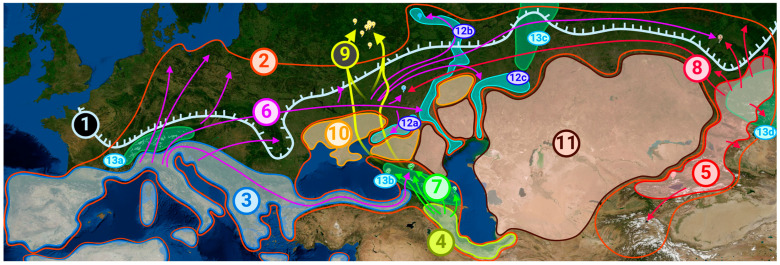
Map of the historical formation of the modern white poplar region in the postglacial era. Lines, areas, arrows, and numbers: pale blue (1)—glacier boundary during maximum glaciation; orange (2)—boundaries of the modern natural range of the white poplar; blue (3)—Southern European refugium; bright green (4)—Transcaucasian refugium; red (5)—Altai–Middle Asian refugium; purple arrows (6)—migration routes from the Southern European refugium to the European part of Russia and then eastward to Siberia, as well as to the Caucasus through northern Turkey; dark green arrows (7)—settlement of the Caucasus Mountains from the Transcaucasian refugium; pink arrows (8)—paths of local range expansion, as well as global migration westward from the Altai–Middle Asian refugium; yellow arrows (9)—artificial settlement of the Central Russia regions (Moscow and its surroundings) by mostly artificially bred poplars on the basis of European and Caucasian genotypes; yellow-orange (10)—steppes (in Pre-Caucasus and southern Ukraine), which are a natural barrier to poplar settlement; brown (11)—semi-deserts and deserts (in Kazakhstan and other Central Asian countries), where white poplars do not live; bright blue (12)—river valleys, where white poplars settle (even if they are surrounded by steppe or desert): 12a—Don, 12b—Volga, 12c—Ural; mint (13)—mountain systems, which were important for white poplar spread: 13a—Alps, 13b—Caucasus, 13c—Ural, 13d—Altai. Color coding of labels: yellow-orange—Central Federal District (Moscow and Moscow region, Obninsk, and Tula are collectively referred to as Center-sX, where X is the unique sample identifier); blue—Uryupinsk (Southern Federal District, referred to as South-sX); purple—Nizhny Novgorod (Volga Federal District, referred to as Volga-sX); yellow-green—Sochi (Southern Federal District, referred to as Caucasus-S-sX); green—Pyatigorsk and surroundings (North Caucasian Federal District, referred to as Caucasus-P-sX); gray-green—Republic of Dagestan (North Caucasian Federal District, referred to as Caucasus-D-sX); brown—Novosibirsk (Siberian Federal District, referred to as Siberia-sX); red—Almaty and surroundings (Kazakhstan, referred to as Kazakhstan-sX). This figure in higher resolution can be found in the Supplementary Materials (Figure S3). Moreover, Figure S4 shows the labels corresponding to our samples alone.

Based on our findings and all of the above information, as well as on the data on the current distribution of white poplar, we formulated a hypothesis of how the formation of its modern range occurred, which is shown in Figure 3; the numbers in this section of the text coincide with the numbers in the figure for ease of perception.

In the late Pliocene, about 2.5 million years ago, a single population of white poplar probably existed across vast territories of Eurasia. However, after a series of glaciations in the Pleistocene, this tree became extinct in a significant part of its historical range, surviving, among other places, in three regions in the territory of Russia and neighboring countries. These regions, those we consider as refugia, were never covered by glaciers and correspond to putative ancestors A1, A2, and A3. The first (5) is the Altai Region and the mountains of Central Asia, located in present-day Russia, Kazakhstan, China (Xinjiang), Kyrgyzstan, Uzbekistan, Tajikistan, and Afghanistan, which matches A1. The second (4) is Transcaucasia (since the Caucasus Mountains themselves were covered by glaciers at that time, as were the southern coast of the Caspian Sea on the territory of modern Azerbaijan, Armenia, eastern Turkey, and northern Iran), and it conforms to ancestor A2. The third region (3) was in the north of Africa and the south of Europe on the Iberian, Apennine, and Balkan peninsulas; on some large islands, including Sicily, Sardinia, and Crete; and on peninsular Asia Minor, settling in the territory of modern Spain, France, Italy, Croatia, Greece, Turkey, and some other countries; this corresponds to ancestor A3. We named these regions the Altai–Middle Asian refugium, the Transcaucasian refugium, and the South European refugium, respectively.

When glaciers began to retreat, white poplars started to spread outside these refugia. It was the third, Southern European refugium that became the most important for the formation of poplars in the European part of Russia. Migrations (6) from it in the northeastern direction led to the settlement of a significant part of the East European Plain, including the vicinities of Nizhny Novgorod and Uryupinsk. At the same time, steppes (10) in the south of modern Ukraine and the Pre-Caucasus, as well as semi-deserts and deserts (11) in the territory of modern Russia, Kazakhstan, and other countries, became a natural obstacle to the spread of the poplar in certain directions. It penetrated the respective regions only along river valleys (12), such as the Don (12a), Volga (12b), and Ural (12c). From the European part of Russia, the poplar migrated further eastward to Western Siberia, making a certain contribution to the gene pool of this region, as can be seen from the results of the genomic analysis of samples collected near Novosibirsk in Figure 3. In parallel with this migration, plants from the Black Sea coastline of Turkey reached the Caucasus when the glaciers retreated from here. This event had an impact on the local gene pool, as some of the Caucasian samples are also carriers of European genes.

The main contribution to the gene pool of modern populations of white poplar in the Caucasus comes from the Transcaucasian refugium. When the glacier in the Caucasus receded, trees from Transcaucasia penetrated northward (7) but did not spread further due to the steppe and semi-desert zones north of the Caucasus Mountains.

The Altai–Middle Asian refugium may have expanded somewhat (8) after the end of glaciation; the poplars living there are the ancestors of modern Chinese plants and, to a large extent, the poplars of Western Siberia. They also migrated westward, at least to the East European Plain, since all plants collected in the vicinity of Uryupinsk are carriers of Altai genes.

We separately note that although the plants we collected in the Central Federal District (Moscow and Moscow Region, Obninsk, Tula, etc.) are genetically close to other European populations and also have a certain percentage of Caucasian genes; in these regions, poplars appeared as a result of introduction in the nineteenth and twentieth centuries (9), so this part of their habitat cannot be called natural.

All pyramidal poplars, two of which were collected by us in Kazakhstan and two in Central Russia, are artificially bred based on European and Caucasian genotypes and are genetically relatively close to each other.

Therefore, we consider Southern Europe, Transcaucasia, and the Altai–Middle Asian system to be potential refugia where the poplar could have survived during glaciations. During the glacial retreat, the poplar spread to regions suitable for it in terms of climatic conditions. This probably happened repeatedly. Our study is the first hypothesis about the formation of the modern range of white poplar. Further research in this direction involving more data is required.

## 4. Materials and Methods

### 4.1. Plant Material

A collection of samples was obtained from 36 trees, representing a range of geographical conditions, for the purposes of the study. The study encompassed a multitude of regions within the European part of the Russian Federation, including the Central Federal District, Volga Federal District, Southern Federal District, and North Caucasus Federal District. Additionally, two specimens were procured from the Republic of Kazakhstan, and three were obtained from the collection of the Central Siberian Botanical Garden of the Siberian Branch of the Russian Academy of Sciences (CSBS SB RAS), situated in the vicinity of the city of Novosibirsk. Detailed information about the samples, including the growing coordinates of each tree, can be found in Table S1; this is also shown in Figure 3 and Figure S4. To ensure accurate sex determination, samples were collected during the flowering period of poplars. A total of 3–10 samples were collected from each region mentioned.

Branches were cut from the trees and transported for two days before DNA extraction. Leaf material for DNA isolation was collected in laboratory conditions and frozen in 2 mL Eppendorf tubes at −70 °C.

### 4.2. DNA Isolation, Library Preparation, and Whole-Genome Sequencing

For DNA isolation, 0.2 g of leaf material was homogenized using a MagNA Lyser automated homogenizer (Roche, Switzerland) in 1 mL of lysis-modified CTAB buffer (100 mM Tris-HCl, pH 8.0; 3% CTAB; 3 M NaCl; 20 mM EDTA, pH 8.0; 1% PVP K30) with 5 μL of β-mercaptoethanol and solid beads. The procedure was similar to that previously described [41], with minor modifications. The homogenate was incubated at 65 °C for 1 h, stirring every 20 min. Two consecutive chloroform purifications were then performed: an equal volume of chloroform was added to the homogenate and centrifuged for 15 min at 10,000× *g* and 20 °C. The aqueous phase was transferred to clean tubes; two volumes of 96% alcohol were added to precipitate DNA. They were incubated for 60 min at −20 °C and then centrifuged for 20 min at 10,000× *g* and 4 °C. The supernatant was carefully removed without touching the precipitate. The precipitate was washed three times with 70% alcohol and centrifuged for 5 min at 10,000× *g* and 4 °C. DNA was quantified using a Qubit^®^2.0 fluorometer (Thermo Fisher Scientific, Waltham, MA, USA); quality control was performed on a NanoDrop^®^ ND−1000 spectrophotometer (NanoDrop Technologies Inc., Wilmington, DE, USA).

The A260/A280 ratio in the DNA samples was 1.8–2.0. Approximately 500 ng of genomic DNA was fragmented into 400 bp double-stranded fragments using the Covaris S220 system (Covaris Inc., Woburn, MA, USA). A double-stranded DNA library was prepared using the VAHTS Universal Plus DNA Library Prep Kit for Illumina V2 (Nanjing Vazyme Biotech Co., Red Maple Hi-tech Industry Park, Nanjing, PRC), according to the manufacturer’s recommendations. AMPure XP beads were used to select DNA libraries by size (500–600 bp). Samples were quantified at standard concentration dilution to 4 nM using a Qubit^®^2.0 fluorometer (Thermo Fisher Scientific, Waltham, MA, USA) and verified on an Agilent 2100 Bioanalyzer using the High Sensitivity DNA Kit (Agilent Technologies, Santa Clara, CA, USA). Genomic DNA of bacteriophage PhiX was used as an internal control. Sequencing was performed using the NextSeq 500/550 High Output Kit v2.5 (300 cycles, 150 + 150 reads) on the Illumina NextSeq 500 platform (Illumina, San Diego, CA, USA) according to the manufacturer’s instructions.

### 4.3. Data Analysis

The analysis was based on genomic profiling using the obtained WGS data. First, reads were trimmed, and residual adapters were removed with Trimmomatic [42]. The trimmed reads were mapped to the *Populus alba* reference genome (GCF_005239225) using bowtie2 [43] in end-to-end mode with parameters adjusted for increased sensitivity (-D 20 -R 3 -N 0 -L 20 -i S,1,0.50). For most samples (61 of 74, including those from NCBI SRA), more than 85% of reads were mapped. However, the ratio of multimapped reads was high enough (about 50% of mapped reads).

The population admixture analysis, principal component analysis (PCA), and phylogenetic tree inference were based either on the genotype likelihood analysis (‘beagles’ derived from BAMs) or SNP profiling. These approaches gave similar results. For these purposes, we used PCAngsd, NGSAdmix, and ‘admixture’ tools [44,45,46]. For SNP calling, we used freeBayes [47], with parameters adjusted for low-coverage data (min. VAF, 0.2; min. mapping Q, 20; min. base Q, 20; min. alternate allele coverage, 4; min. summary coverage, 5). One million SNPs with Phred Quality > 25 were randomly selected for the analysis. Among all found SNPs, 18% were indels.

Also, we inferred UPGMA trees (with 500 bootstraps) based on the Euclidean pairwise distance matrix for per-sample variant allele frequency (VAF) data. As an outgroup, we used either *Salix pentandra* (WGS data were obtained from the NCBI SRA, run ID SRR16779480, PRJNA775003) or *Populus trichocarpa* (GCA_000002775.4; reads were simulated with the wgsim tool).

Additionally, we inferred bootstrapped maximum likelihood (ML) phylogenetic trees. As the sequencing depth was insufficient (5×–20×) for obtaining high-quality genomic assemblies, we derived a pseudo-multiple sequence alignment (MSA) based on SNP calling data using the vcf2phylip tool [48]. We restricted the analysis to the coding regions from the *P. alba* GCF_005239225 reference assembly to reduce noise levels and optimize computational costs. MSA was post-filtered with the trimAl tool [49]. Additionally, we retained only MSA positions with fewer than 4 Ns. Phylogenetic tree inference was performed using RAxML-NG [50] and IQ-TREE [51]. The optimal evolutionary model was selected using the IQ-TREE ModelFinder module (TVMe + R4 was chosen as optimal). We used normalized Robinson–Foulds metrics (using the TreeDist package) to compare tree topologies obtained using various models (JC, GTR + I + G, TVMe + R4, and others), as well as the results derived with IQ-TREE and RAxML-NG. The differences were negligible (normalized RF dist < 0.15 for all cases). The final ML phylogenetic tree was derived with RAxML-NG using 500 bootstrap replicates, since IQ-TREE UF-bootstrap values tend to be overestimated.

Also, we applied another approach to infer ML trees, using the read2tree [52] tool with 2000 marker orthologous genes (OMAs) from the OMA browser [53] present in *Populus trichocarpa*, *Ricinus communis*, *Manihot esculenta*, and *Quercus lobata*. However, the resulting ML trees were very sensitive to the quality of the source data: (1) samples with a high duplication rate of raw reads and/or low BUSCO [54] completeness of the corresponding SPAdes [55] assemblies were clustered together (even after we removed samples with the lowest coverage and MSA positions containing any Ns or gaps). Also, (2) we observed pronounced batch effects, which did not appear in phylogenetic trees described above (SNP-based or genotype likelihood-based). This trend was reproduced when analyzing subsets of 500 OMAs, independently of ML tree inference tools (RAxML-NG [50] and IQ-TREE [51]) and substitution models.

The population admixture analysis was performed using NGSAdmix, ‘admixture’, and PCAngsd tools [44,45,46], with K values ranging from 2 to 6. The results based on SNPs and genotype likelihood analysis were quite similar, if discounting variations at K > 3, when different tools or initial seeds were used. PCAngsd was also used to perform PCA. A cross-validation procedure was performed by the ‘admixture’ tool in order to pick up the optimal K. Also, the variation in the predicted structures (derived from various seeds) was evaluated in terms of mean (across all samples) per-sample deviation of ancestor ratios from the mean value across all seeds (per sample). Ancestors were pre-sorted by the average ratio among all the samples. However, unlike CV error estimation, this indirect approach for selecting optimal K is not commonly accepted and may be biased, and it should be used only as a hint.

To increase the geographic coverage of the analysis, additional samples of white poplar trees were included, grown in Italy and Xinjiang Province of China. The WGS data were obtained from the NCBI SRA (accession IDs are presented in the Supplementary Table S1). These data correspond to several research articles [56,57,58,59,60].

## 5. Conclusions

In this study, using a genomic approach and data from different regions of Russia, as well as Italy, Kazakhstan, and China, we proposed a hypothesis about the formation of the modern range of white poplar. We hypothesize that before the glaciation period, the range of *Populus alba* was more unified and then divided into three refugia, South European, Transcaucasian, and Altai–Middle Asian, which indicates that the poplar survived in places not affected by glaciers. After glacial retreat, populations from these refugia probably became the basis for modern poplar populations in Europe, the Caucasus, and Siberia, which then spread and regained range integrity. However, further studies aimed at increasing the number of specimens and expanding study areas are needed to more reliably confirm and refine this hypothesis, as they are still preliminary.

## Data Availability

All data obtained in our study are in the public domain. Whole-genome sequencing results for 36 *P. alba* individuals are available at the NCBI under an accession number at https://www.ncbi.nlm.nih.gov/bioproject/PRJNA1109753 (accessed on 1 September 2024).

## Supplementary Materials

The following supporting information can be downloaded at https://www.mdpi.com/article/10.3390/plants14213328/s1: Figure S1. Bootstrapped UPGMA phylogenetic tree based on SNP data (Euclidean distance matrix for per-sample VAF vectors). Figure S2. Bootstrapped maximum likelihood (ML) tree based on SNP data for coding regions (pseudo-MSA; RAxML). Figure S3. Map of the historical formation of the modern white poplar region in the postglacial era (the same as Figure 3 in the main text, but in high resolution). Figure S4 shows the labels corresponding to geographical locations at which the samples were collected, with colors identical to Figure 3 (main text). Figure S5. SNP-based population admixture analysis using NGSAdmix with K = 2, 3, 4, 5, 6. Figure S6. SNP-based population admixture analysis using ADMIXTURE with K = 2, 3, 4, 5, 6. Figure S7. SNP-based population admixture analysis using PCAngsd with K = 2, 3, 4, 5, 6. Figure S8. Cross-validation errors with various K values (produced by ‘admixture’). Table S1 lists the sampled poplar trees, their labeling during the study, coordinates of growth, and assigned sex. Table S2 contains information about reference *P. alba* genomes used in our study.
