# Supplementary figures and images for: Genomic Data Suggests Pathways of Modern White Poplar (Populus alba L.) Range Formation in the Postglacial Era
Source: Plants (Basel). 2025 Oct 30;14(21):3328. doi: 10.3390/plants14213328 (PMC12608353; doi:10.3390/plants14213328)

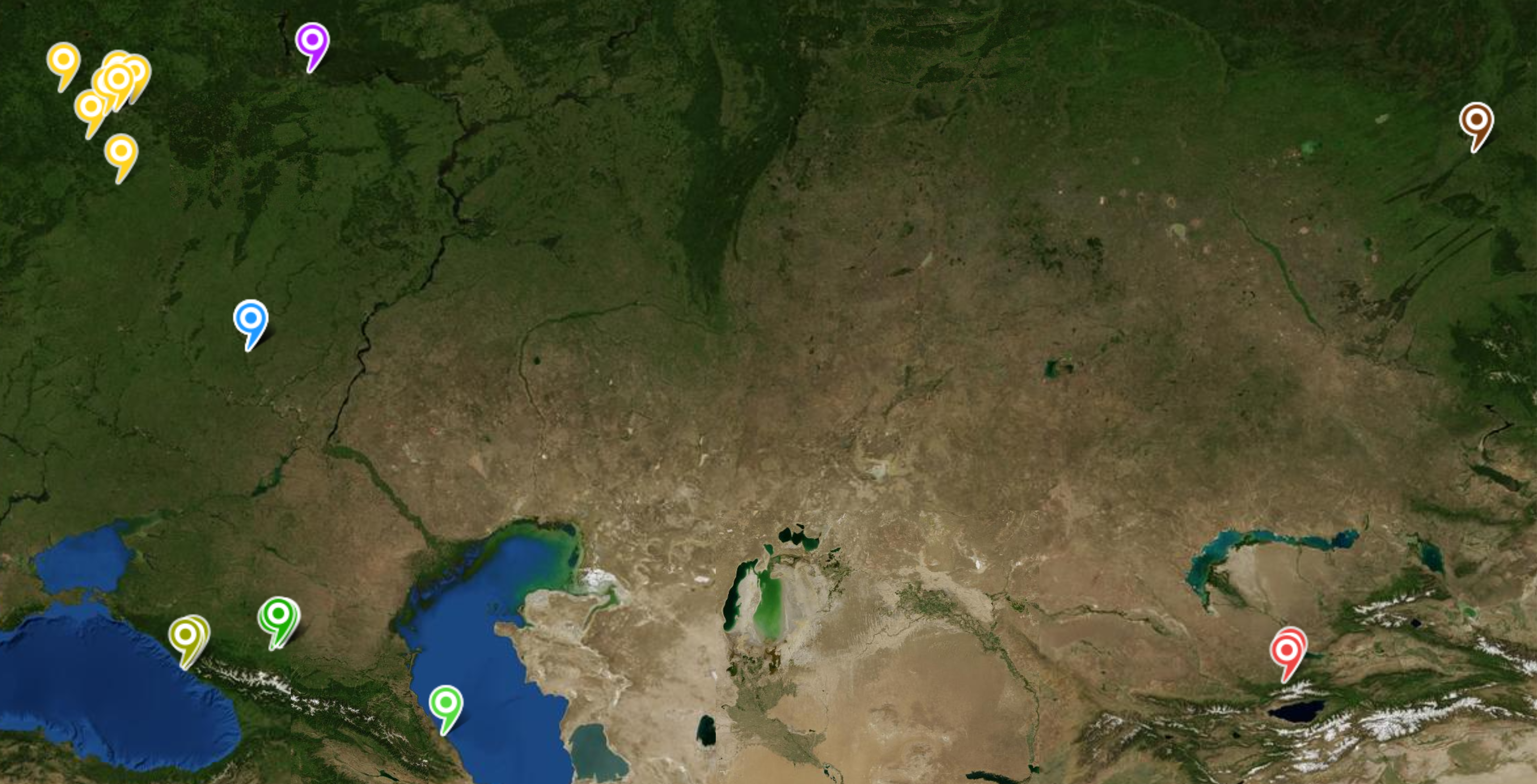

Supplement: Supplementary file 1 [file plants-14-03328-s001.zip › Figure_S3.png]

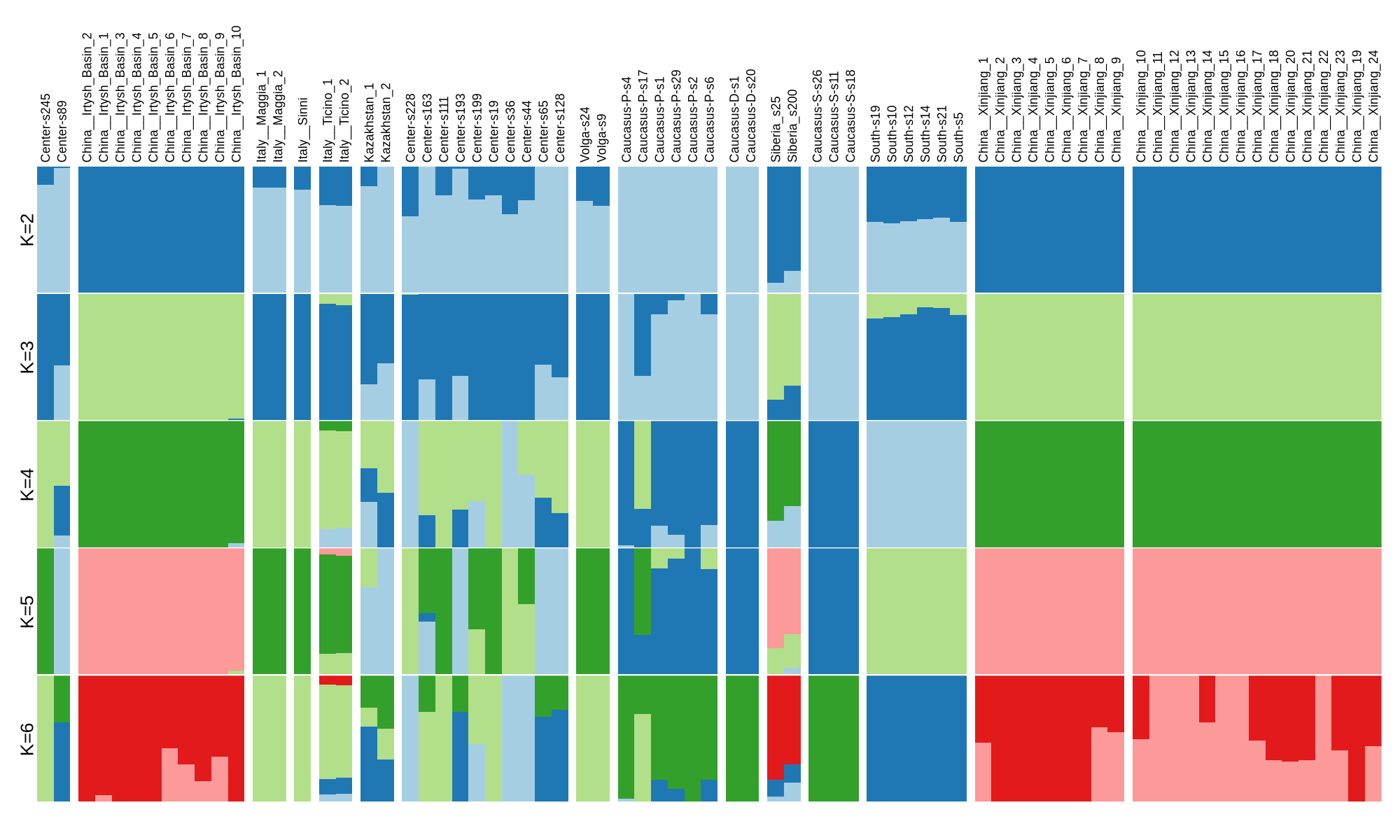

Supplement: Supplementary file 1 [file plants-14-03328-s001.zip › Figure_S4.png]

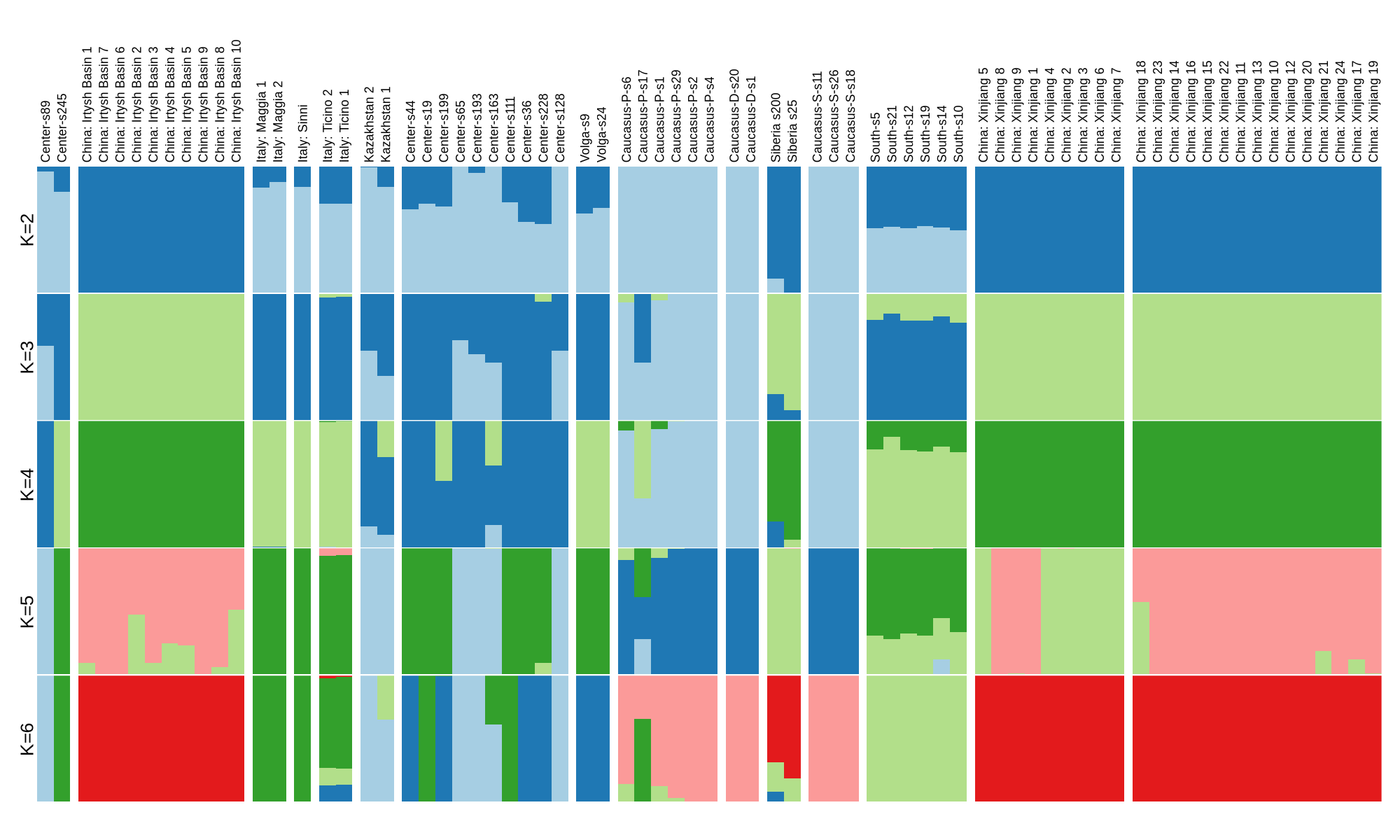

Supplement: Supplementary file 1 [file plants-14-03328-s001.zip › Figure_S5.png]

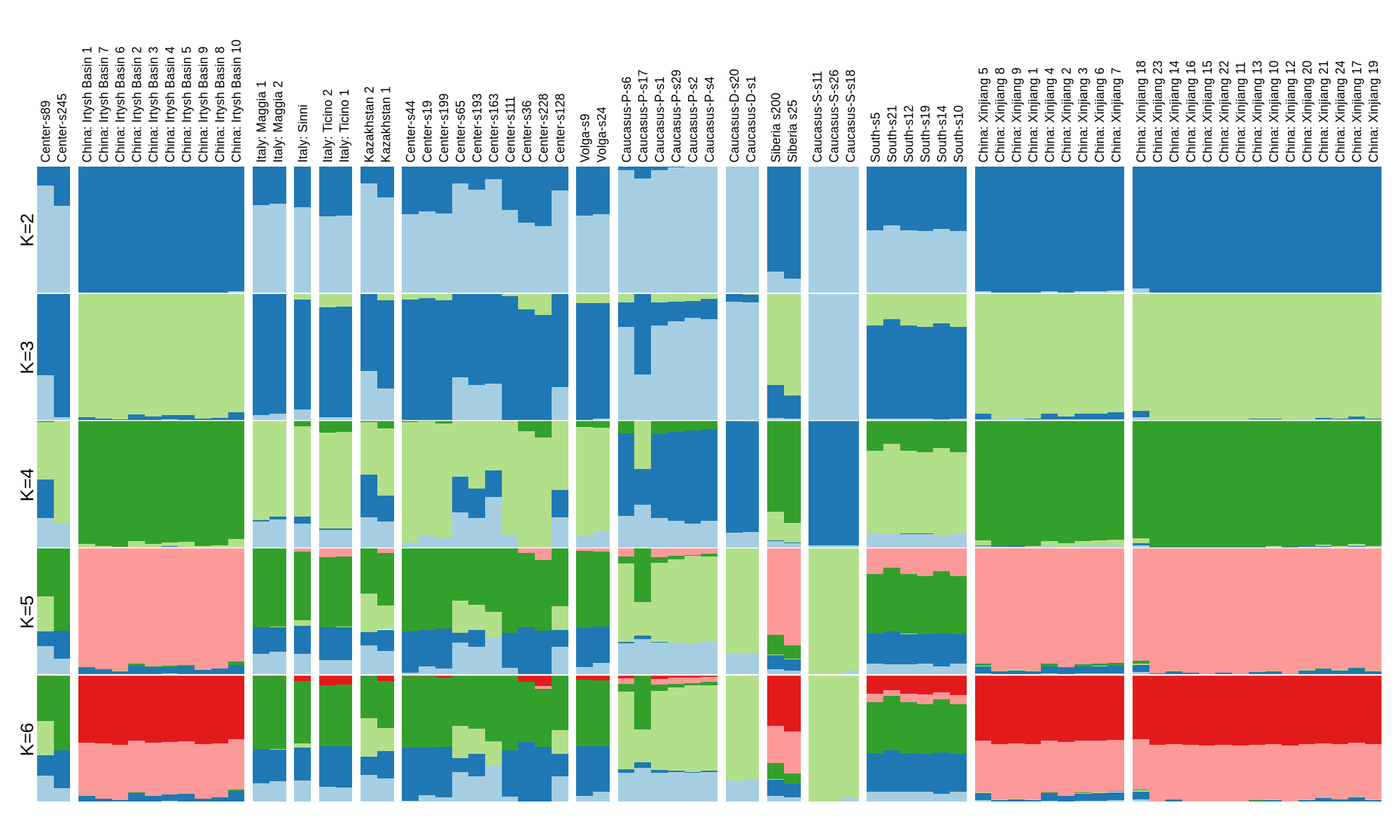

Supplement: Supplementary file 1 [file plants-14-03328-s001.zip › Figure_S6.png]

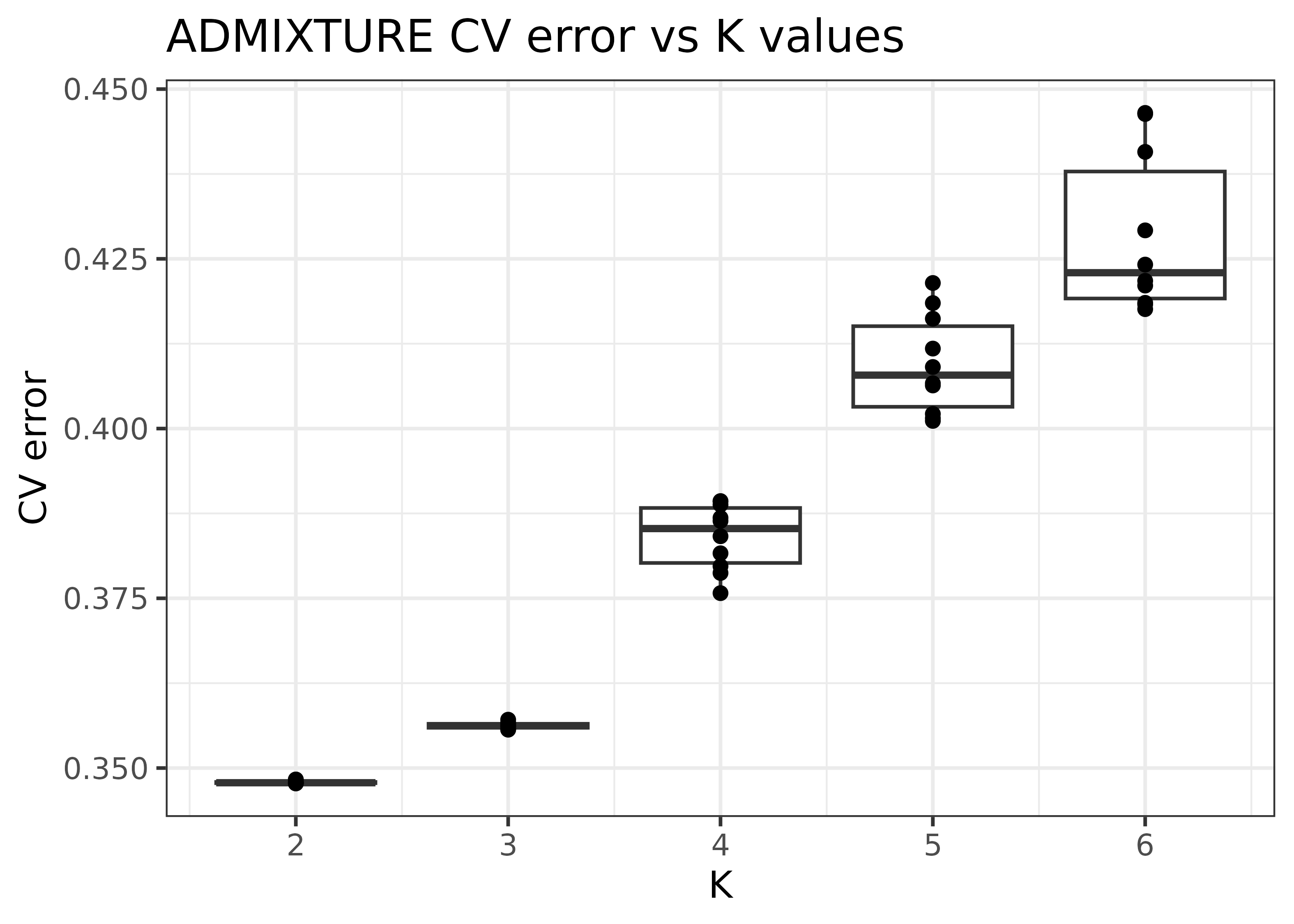

Supplement: Supplementary file 1 [file plants-14-03328-s001.zip › Figure_S7.png]
